# Burden of Disease in Coastal Areas of South Korea: An Assessment Using Health Insurance Claim Data

**DOI:** 10.3390/ijerph16173044

**Published:** 2019-08-22

**Authors:** Munkhzul Radnaabaatar, Young-Eun Kim, Dun-Sol Go, Yunsun Jung, Seok-Jun Yoon

**Affiliations:** 1 Department of Public Health, Graduate School, Korea University, Seoul 02841, Korea; 2 Department of Preventive Medicine, Korea University College of Medicine, Seoul 02841, Korea

**Keywords:** coastal area, South Korea, population health, burden of disease

## Abstract

Background: While measuring and monitoring disease morbidity, it is essential to focus on regions experiencing inequitable health outcomes, especially coastal populations. However, no research investigating population health outcomes in coastal areas has been conducted. Therefore, we aimed to investigate the burden of disease morbidity in coastal areas of South Korea. Methods: Using an administrative division map and the ArcGIS, we identified and included 496 coastal districts. In this observational study, years lived with disability (YLDs) were estimated using incidence-based approaches to calculate the burden of disease in 2015. Incidence and prevalence cases were collected using National Health Insurance Service (NHIS) medical claim data using a specialized algorithm. Results: Age-standardized years lived with disability (ASYLDs) in the coastal areas were 24,398 per 100,000 population, which is greater than the 22,613 YLDs observed nationwide. In coastal areas, the burden of disease morbidity was higher in females than in males. Diabetes mellitus was the leading specific disease of total YLDs per 100,000 population, followed by low back pain, chronic obstructive pulmonary disease, osteoarthritis, and ischemic stroke. Conclusion: In this study, the coastal areas of South Korea carry a higher burden than the national population. Additionally, chronic diseases compose the majority of the health burden in coastal areas. Despite the limitation of data, YLD was the best tool available for evaluating the health outcomes in specific areas, and has the advantage of simplicity and timely analysis.

## 1. Introduction

Health, which can be quite complex and subjective, is measured using many methods [1]. One efficient method for accomplishing the measurement of health outcomes is the use of disability-adjusted life years (DALY). DALY is a result of the Global Burden of Disease (GBD) project, a collaboration between the WHO and the World Bank, and is evaluated and periodically improved by the Institute for Health Metrics and Evaluation (IHME) [2]. DALY is single unit that measures disease burden due to morbidity and mortality. The unit allows comparisons between countries and at subnational levels, and also provides information regarding the health status of a population [3]. DALY is the sum of years lived with disability (YLDs) and years of life lost (YLLs). The YLDs indicate the impact of disease morbidity, which means that the larger the YLDs, the larger the burden of disease due to morbidity. Since the development of DALY, the concept has been adopted by many countries and is calculated using epidemiological data, health surveys, and published research [4]. In South Korea several studies have been conducted to estimate DALYs using health surveys [5] and medical claim data [6,7]. However, no previous research has been conducted to investigate the burden of disease in a particular geographic area. To elaborate, health outcome in coastal areas is rarely discussed, considering its importance.

Being a longstanding epidemiological concern, the place of residence is an important factor in analysis [8]. The coastal areas are adjacent to the sea and have access to various marine resources that have health benefits, such as seaweeds, sea waters, and ocean breeze. Studies from England and Australia show that people who live in coastal areas reported better health than those living further inland [9,10,11]. However, contradictory findings were reported in South Korea. To elaborate, the rate of chronic disease among the elderly residents of farming-fishing villages were twice higher than those in urban communities [12]. South Korea has experienced rapid demographic changes due to increasing life expectancy and declining fertility rates. In 2018, South Korea earned the status of an aged society, as defined by the World Health Organization (WHO) and the United Nations [13]. With an aging population comes specific concerns regarding the rising cost of medical expenditures as well as a shift in the patterns of medical conditions [14]. Therefore, effective healthcare programs should be established, and long-term countermeasures against disease must be prepared. Given this, it is essential to measure and monitor the burden of disease morbidity in Korea, especially in the coastal area populations. Thus, the purpose of this study was to measure the burden of disease morbidity in coastal areas of South Korea using YLDs.

## 2. Methods

The following steps were completed to estimate the burden of disease morbidity in coastal areas. First, we defined the study population. Second, a dataset of incidence, prevalence, mortality, and case fatality was established according to specific diseases, sex, and age group. Third, the burden of each disease was calculated using the aforementioned estimated variables.

### 2.1. Study Population

We defined coastal areas as the administrative divisions nearest to the boundary of the shoreline. We selected these areas using an administrative division map and the ArcGIS program (ESRI 2011. ArcGIS Desktop: Release 10. Redlands, CA, USA: Environmental Systems Research Institute). The South Korean administrative districts were composed of eight metropolitan cities and nine provinces, which included a total of 3500 towns, townships, and neighborhoods in 2015 [15]. We identified 496 coastal administrative divisions from among the coastal towns, townships, and neighborhoods. We grouped these administrative districts by city- and province-administration level by administrative agency code. The coastal areas of Busan metropolitan city, Incheon metropolitan city, Ulsan metropolitan city, Gyeonggi province, Gangwon province, Chungcheongnam province, Jeollabuk province, Jeollanam province, Gyeongsangbuk province, Gyeongsangnam province, and Jeju province were included in our study.

### 2.2. Data Collection 

The number of incident and prevalent cases were collected using claim reimbursement data from the National Health Insurance Service (NHIS) medical utilization database, which contained the data of 52 million individuals in 2015. We calculated the prevalence and incidence of 216 specific diseases (excluding injury cases) using previously developed algorithms based on ICD-10 code. The algorithms were previously developed in the Korean Burden of Disease Study. In order to prevent overestimation for each disease, the authors extracted the number of times hospitalization occurred and the number of outpatient visits (OP 1–5) in a fixed period based on literature review and consulted experts on the feasibility of the study. To estimate incidence, washout period (WP) was applied (1–5 years) [16]. WP was set to isolate new cases in the present year from new cases in previous years. For example, the prevalence of dental caries was calculated as the number of patients with dental caries who were assigned K02 as a primary diagnostic code more than 3 times during outpatient visits or at hospital admissions within a 1-year period. To calculate incidence, we set the 5-year period before 2013 as a washout period. Therefore, patients were included as incident cases for a given disease if the following two conditions were satisfied: (1) No usage of a given disease code as a primary diagnostic code from 2008–2012 and (2) the prevalence criteria (e.g., the primary diagnostic code was assigned more than 3 times) was satisfied in 2013. We used the cause of death data from the National Statistical Office to generate case fatality rate and mortality rate. To estimate case fatality rates, the number of deaths due to each disease were divided by the number of incident cases. 

### 2.3. Estimate Years Lived with Disability

We used an incidence-based approach to measure YLDs. The following formula further explains the measurement [2,17].

(1) $$\begin{array}{l} YLD=D\{\frac{KCe^{\gamma \alpha }}{{\left(\gamma +\beta \right)}^{2}}[e^{-\left(\gamma +\beta \right)\left(L+\alpha \right)}\left[-\left(\gamma +\beta \right)\left(L+\alpha \right)-1\right]\\ \;-e^{-\left(\gamma +\beta \right)\alpha }\left[-\left(\gamma +\beta \right)\alpha -1\right]]+\frac{1-K}{\gamma }\left(1-e^{-\gamma L}\right)\} \end{array}$$

where γ is discount rate, β is the age-adjusted parameter (=0.04), *K* is 1 or 0 (modulation factor), *C* is constant (=0.1658), *α* is age at disease onset, *L* is mean duration, and *D* is disability weight.

The following variables were required for estimating YLDs: Incidence of a particular disease, mean duration, age at onset, and disability weight (DW). Previously, the number of incident cases was calculated by sex and one-year age group along with prevalence, mortality, and case fatality. The information on the duration of the disease and age of onset was not available; thus, we used the software package called “DisMod II,” which was made available to the public domain by the World Health Organization. DisMod II was used for analyzing the mean duration and age of onset of each disease from previously estimated prevalence, incidence, case fatality, and mortality [18]. We applied the DW results developed in the 2012 Korean Burden of Disease Study. The DWs of major diseases and the calculated results of DWs are published elsewhere [19].

All age YLDs and age-standardized years lived with disability (ASYLDs) were used as indicators to estimate the burden of disease due to morbidity. In order to calculate the rate of ASYLDs, we used the data of the population registered in 2015 from the Ministry of the Interior. Additionally, the results for ASYLDs at the national level are provided with the consent of the authors of the Korean National Burden of Disease Study 2015 for comparison [20]. 

In this study, 216 specific diseases were classified into 3 levels for analysis. At Level 1, all 216 diseases were classified into 2 categories: Communicable, maternal, neonatal, and nutritional diseases (CMNN), or noncommunicable diseases (NCD). At Level 2, all 216 diseases were classified into 17 disease groups: Cardiovascular and circulatory diseases; chronic respiratory diseases; cirrhosis of the liver; diabetes, urogenital, blood, and endocrine diseases; digestive diseases (except cirrhosis); diarrhea; lower respiratory infections, meningitis, and other common infectious diseases; HIV/AIDS and tuberculosis; maternal disorders; mental and behavioral disorders; musculoskeletal disorders; neglected tropical diseases and malaria; neonatal disorders; neoplasm; neurological disorders; nutritional deficiencies; other communicable, maternal, neonatal, and nutritional disorders; or other noncommunicable diseases. Lastly, Level 3 contained all 216 diseases. 

### 2.4. Ethics Statement

Approval for the study was obtained from the Korea University Institutional Review Board (KU-IRB-2019-0005-01). As this study used secondary data and contained no personal information, the need for informed consent was waived.

## 3. Results

Demographic characteristics are shown in Table 1. A total of 4,710,202 individuals were defined as our study population which is equivalent to 9.1% of the South Korean total population. There were 2,393,120 (50.8%) males and 2,317,082 (49.2%) females, respectively. In the study population, 31.7% were from metropolitan cities and the remaining 68.3% were from provinces. Among them, the coastal area of Busan metropolitan city had the biggest part of the population with 17.1%, followed by Jeollanam Province (16.4%), and Gyeongsangnam Province (16.4%).

As shown in Table 2, in 2015 the total of all age YLDs and ASYLDs caused by the burden of disease in coastal areas was 1,192,657 person-years and 24,398 per 100,000 population, respectively. In contrast, the total of all age YLDs and ASYLDs caused by the burden of disease at the national level was 11,728,668 person-years and 22,613 per 100,000 population, respectively. The burden of disease morbidity in coastal areas was 7.3% higher than the national level per 100,000 population (7.7% and 6.4% higher burden for males and females, respectively). In addition, females contributed more all age YLDs and ASYLDs than males in coastal areas and nationwide. Also, NCD accounted for 97.5% of the total YLDs, and CMNN for the remaining 2.5%.

**Figure 1:**
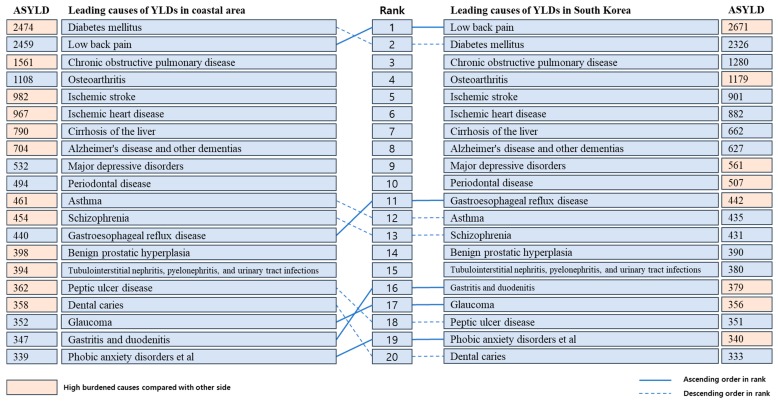
Top 20 leading causes (Level 3) of ASYLDs in the study population and total population, 2015. Abbreviations: ASYLD, age-standardized years lived with disability; YLDs, years lived with disability. Ascending and descending order in rank is based on ASYLD in coastal areas.

The top 20 diseases (Level 3) that caused the highest ASYLDs per 100,000 population in the coastal areas and national level are presented in Figure 1. At a national level, low back pain ranked first (2671 ASYLDs per 100,000 population), followed by diabetes mellitus (DM) (2326 ASYLDs per 100,000 population) and chronic obstructive pulmonary disease (COPD) (1280 ASYLDs per 100,000 population). In contrast, DM caused the greatest burden of disease morbidity with 2474 ASYLDs per 100,000 population in the coastal areas. Low back pain ranked second (2459 ASYLDs per 100,000), followed by COPD (1561 ASYLDs per 100,000 population), osteoarthritis (1107 ASYLDs per 100,000 population), and ischemic stroke (982 ASYLDs per 100,000 population). The top 20 leading specific diseases accounted for 65.98% of all YLDs.

**Figure 2:**
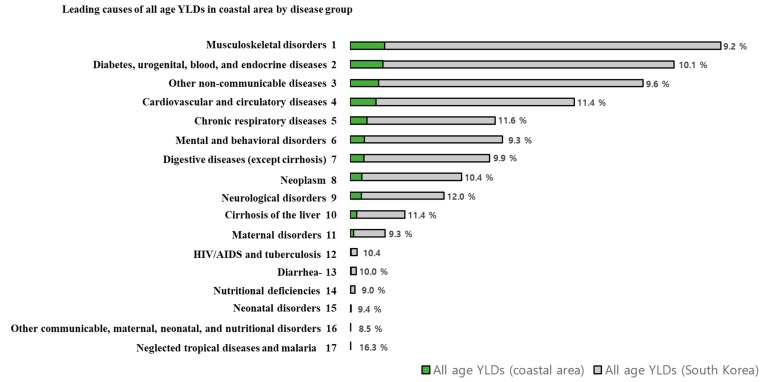
All age YLDs in coastal areas and in South Korea by Level 2 disease group, 2015. Abbreviations: YLDs, years lived with disability. Data refer to the proportion of all age YLDs in the coastal areas among South Korea’s all age YLDs. Sorted by descending all age YLDs in coastal areas.

The burden of disease of the Level 2 disease group is presented in Figure 2, sorted by YLDs in the coastal areas in descending order. The analysis revealed that musculoskeletal disorders contributed the most YLDs in the study population (17.1%), followed by diabetes, urogenital, blood, and endocrine diseases (16.4%), other noncommunicable diseases (14.1%), cardiovascular and circulatory diseases (12.8%), and chronic respiratory diseases (8.4%). The data on the figure refer to the cause-specific percentage of all age YLDs in the coastal areas among the all age YLDs of South Korea. If we compare the percentages displayed on Figure 2, neglected tropical diseases and malaria are ranked first (16.3%), followed by neurological disorders (12.0%), chronic respiratory diseases (11.6%), and cirrhosis of the liver (11.4%). 

**Figure 3:**
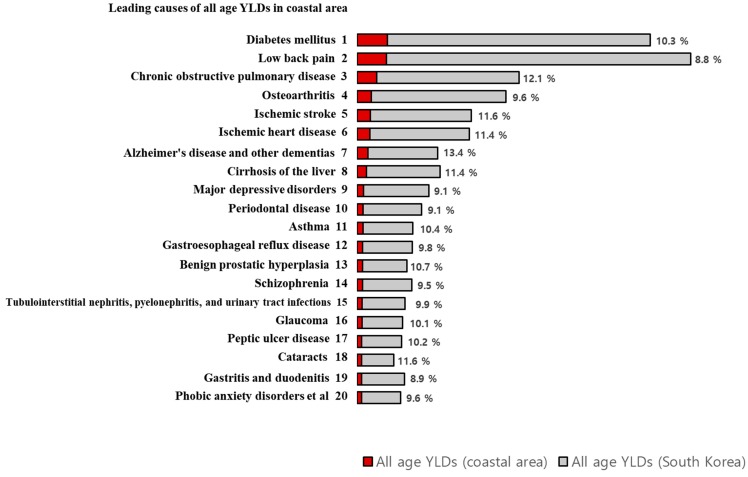
All age YLDs in coastal areas and in South Korea by Level 3 disease group, 2015. Abbreviations: YLDs, Years lived with disability. Data refer to the proportion of all age YLDs in the coastal areas among South Korea’s all age YLDs. Sorted by descending all age YLDs in coastal areas.

Additionally, Figure 3 displays the top 20 contributors (Level 3) to the burden of disease morbidity in the coastal population along with all age YLDs in the national population. The data on the figure refer to the percentage of YLDs in the coastal areas compared to the YLDs of South Korea as a whole. In our study population, DM contributed the greatest burden; however, if we compare the percentages on the figure, Alzheimer’s disease and other dementias ranked first (13.4%), followed by COPD (12.1%), ischemic stroke (11.6%), and cataracts (11.6%). As for the male population in the coastal study group (data not shown), Alzheimer’s disease and other dementias placed first (12.9%) and COPD second (12.6%), followed by hypertensive heart disease (12.4%), and cirrhosis of the liver (11.7%). In females, Alzheimer’s disease and other dementias placed first (13.6%), followed by ischemic stroke (11.9%), ischemic heart disease (11.7%), cataracts (11.7%), and COPD (11.7%).

## 4. Discussion

This study measured the burden of disease morbidity in coastal areas of South Korea using medical claim data to calculate the YLD. We found that coastal areas had more YLDs than the total population, with 1785 ASYLD per 100,000 population. Additionally, females had more YLDs than males. Although the top 20 leading diseases (Level 3) of burden of disease morbidity in the coastal areas were similar to those at the national level, the ASYLDs per 100,000 population were substantially higher in the coastal population for DM, COPD, ischemic stroke, ischemic heart disease, cirrhosis of the liver, and Alzheimer’s disease.

To the best of our knowledge, there has been no previous study about population health measurement in a coastal area. However, a few studies investigating the medical utilization of rural and fishing areas have been published. For example, Yu and Kim (2010) examined the disease conditions of patients from a farming and fishing area using medical records of 2365 discharged patients. They found that the top five groups of diseases were: Diagnoses of injury, poisoning, and certain other consequences of external causes (18.4%); diseases of the respiratory system (15.5%); diseases of the circulatory system (11.5%); diseases of the digestive system (11%); and neoplasms (10.3%) [12]. However, in our study, the burden of musculoskeletal disorders ranked first, followed by diabetes, urogenital, blood, and endocrine diseases, other noncommunicable diseases, cardiovascular and circulatory diseases, and chronic respiratory diseases. Yu and Kim’s findings are from only one hospital, and therefore, results cannot be generalized for the entire coastal population. In contrast, our study is meaningful in that we present the level of health status in coastal areas using a large dataset.

Figure 2 and Figure 3 present the proportion of all age YLDs in the coastal areas compared to those of the total all age YLDs in South Korea by Level 2 and 3 disease groups. For each disease, we expected that the proportion of YLDs of the coastal areas would be more than 10.2% (which is the percentage of the disease burden of South Korea as a whole). However, in coastal areas at Level 3, the following diseases contributed fewer YLDs than expected: Low back pain, osteoarthritis, major depressive disorders, periodontal disease, gastroesophageal reflux disease, schizophrenia, tubulointerstitial nephritis, pyelonephritis, urinary tract infections, gastritis and duodenitis, and phobic anxiety disorders. For males, low back pain, periodontal disease, glaucoma, schizophrenia, and major depressive disorders contributed fewer YLDs than expected. Among females, low back pain, osteoarthritis, major depressive disorders, gastroesophageal reflux disease, periodontal disease, abortion, gastritis and duodenitis, dental caries, and phobic anxiety disorders contributed fewer YLDs compared with the expected YLDs. Based on this, therefore, we presume that the coastal areas may have potential health benefits related to the diseases listed herein. The coastal areas are adjacent to the sea and have various marine resources with significant health benefits, such as seaweeds, sea water, and ocean breeze. Several studies have been conducted to identify the relationship between health behaviors and quality of life in coastal areas and the marine resources [9,10,11]. Alternatively, YLDs in coastal areas may be underestimated due to a lack of medical resources [21]. According to the Statistical Yearbook of Health Insurance [22], 52.8% of the doctor population was active in Seoul, Incheon, and Gyeonggi Province and thereby concentrated around the capital. Moreover, poor access to medical care may result in unmet needs in coastal areas. Further research is needed to determine whether these gaps are attributable to differences in medical accessibility or socio-economic status. 

Despite the importance of capturing the health outcomes in coastal areas, a lack of available data has previously prevented health-outcome research in specific regions. However, in this study we were successful in estimating the burden of disease in these regions, largely due to South Korea’s health insurance claim data. It is likely that other countries with more complicated healthcare systems face greater challenges in estimating the burden of disease morbidity [23]. Based on our objective and the data available to us, YLD was the best available tool for evaluating the health outcomes of the coastal divisions, which has the advantage of simplicity and makes it possible to analyze health outcomes using large datasets.

A limitation to our study is that the NHIS medical claim database was not established for research purposes. Thus, it is possible that the epidemiological parameters derived from claim data may not be completely acceptable from a clinical perspective. Second, we excluded injuries in our study due to limitations in connecting databases by administration level. We assume that inclusion of injuries will show significant differences in ranking. Third, we did not consider urban and rural administrative districts within coastal regions and in South Korea. However, future studies can overcome these shortcomings by expanding the range of objectives.

## 5. Conclusions

In this study, the coastal areas in South Korea were found to carry a higher burden than the national population. Additionally, chronic diseases compose the majority of the health burden in coastal areas. Despite the limitation of data, YLD was the best tool available for evaluating the health outcomes in this specific area, as it has the advantage of simplicity and timely analysis. 

## Figures and Tables

**Table 1:** Characteristics of study population.

Characteristic	Total Population (*N* = 51,529,338)	Study Population *(N* = 4,710,202 (9.1%))
***Age (years)***		
0–9	4,601,688 (8.9%)	414,289 (8.8%)
10–19	5,717,089 (11.1%)	478,909 (10.2%)
20–29	6,699,048 (13.0%)	529,236 (11.2%)
30–39	7,670,966 (14.9%)	650,427 (13.8%)
40–49	8,858,993 (17.2%)	756,879 (16.1%)
50–59	8,324,791 (16.2%)	785,710 (16.7%)
60–69	5,073,279 (9.8%)	539,527 (11.5%)
70–79	3,176,437 (6.2%)	375,722 (8.0%)
Over 80	1,407,047 (2.7%)	179,503 (3.8%)
***Sex***		
Male	25,758,186 (49.99%)	2,393,120 (50.8%)
Female	25,771,152 (50.01%)	2,317,082 (49.2%)
***Administrative level***		
Metropolitan city		
Seoul	10,022,181 (19.4%)	0 (-)
Busan	3,513,777 (6.8%)	803,694 (17.1%)
Daegu	2,487,829 (4.8%)	0 (-)
Incheon	2,925,815 (5.7%)	484,811 (10.3%)
Gwangju	1,472,199 (2.9%)	0 (-)
Daejeon	1,518,775 (2.9%)	0 (-)
Ulsan	1,173,534 (2.3%)	203,250 (4.3%)
Sejong	210,884 (0.4%)	0 (-)
Province		
Gyeonggi	12,522,606 (24.3%)	428,697 (9.1%)
Gangwon	1,549,507 (3.0%)	278,579 (5.9%)
Chungcheongbuk	1,583,952 (3.1%)	0 (-)
Chungcheongnam	2,077,649 (4.0%)	261,525 (5.6%)
Jeollabuk	1,869,711 (3.6%)	120,827 (2.6%)
Jeollanam	1,908,996 (3.7%)	773,282 (16.4%)
Gyeongsangbuk	2,702,826 (5.2%)	252,944 (5.4%)
Gyeongsangnam	3,364,702 (6.5%)	771,073 (16.4%)
Jeju	624,395 (1.2%)	331,520 (7%)

**Table 2:** YLDs in South Korea and coastal areas, by sex, 2015.

YLDs	South Korea	Coastal Area
***All age YLDs (% of YLD in South Korea)***		
Total	11,728,668	1,192,657 (10.2%)
Male	5,391,824	567,257 (10.5%)
Female	6,336,844	625,400 (9.9%)
***ASYLD (Per 100,000 population)***		
Total	22,613	24,398
Male	20,932	22,683
Female	24,589	26,281

Abbreviations: YLDs, years lived with disability; ASYLD, age-standardized years lived with disability.
